# Macrophages in ovarian cancer and their interactions with monoclonal antibody therapies

**DOI:** 10.1093/cei/uxab020

**Published:** 2021-11-22

**Authors:** Gabriel Osborn, Chara Stavraka, Rebecca Adams, Ahmad Sayasneh, Sharmistha Ghosh, Ana Montes, Katie E Lacy, Rebecca Kristeleit, James Spicer, Debra H Josephs, James N Arnold, Sophia N Karagiannis

**Affiliations:** St. John’s Institute of Dermatology, School of Basic and Medical Biosciences, King’s College London, London, United Kingdom; St. John’s Institute of Dermatology, School of Basic and Medical Biosciences, King’s College London, London, United Kingdom; Department of Oncology, Guy’s and St Thomas’ NHS Foundation Trust, Great Maze Pond, London, United Kingdom; School of Cancer and Pharmaceutical Sciences, King’s College London, London, United Kingdom; St. John’s Institute of Dermatology, School of Basic and Medical Biosciences, King’s College London, London, United Kingdom; Department of Gynecological Oncology, Surgical Oncology Directorate, Guy’s and St Thomas’ NHS Foundation Trust, School of Life Course Sciences, King’s College London, London, United Kingdom; Department of Oncology, Guy’s and St Thomas’ NHS Foundation Trust, Great Maze Pond, London, United Kingdom; Department of Oncology, Guy’s and St Thomas’ NHS Foundation Trust, Great Maze Pond, London, United Kingdom; St. John’s Institute of Dermatology, School of Basic and Medical Biosciences, King’s College London, London, United Kingdom; Department of Oncology, Guy’s and St Thomas’ NHS Foundation Trust, Great Maze Pond, London, United Kingdom; School of Cancer and Pharmaceutical Sciences, King’s College London, London, United Kingdom; St. John’s Institute of Dermatology, School of Basic and Medical Biosciences, King’s College London, London, United Kingdom; Department of Oncology, Guy’s and St Thomas’ NHS Foundation Trust, Great Maze Pond, London, United Kingdom; School of Cancer and Pharmaceutical Sciences, King’s College London, London, United Kingdom; School of Cancer and Pharmaceutical Sciences, King’s College London, London, United Kingdom; St. John’s Institute of Dermatology, School of Basic and Medical Biosciences, King’s College London, London, United Kingdom; Breast Cancer Now Research Unit, School of Cancer and Pharmaceutical Sciences, King’s College London, Guy’s Cancer Centre, London, United Kingdom

**Keywords:** macrophages, antibodies, immunotherapy, tumour immunology, Fc receptors

## Abstract

The unmet clinical need for effective treatments in ovarian cancer has yet to be addressed using monoclonal antibodies (mAbs), which have largely failed to overcome tumour-associated immunosuppression, restrict cancer growth, and significantly improve survival. In recent years, experimental mAb design has moved away from solely targeting ovarian tumours and instead sought to modulate the wider tumour microenvironment (TME). Tumour-associated macrophages (TAMs) may represent an attractive therapeutic target for mAbs in ovarian cancer due to their high abundance and close proximity to tumour cells and their active involvement in facilitating several pro-tumoural processes. Moreover, the expression of several antibody crystallisable fragment (Fc) receptors and broad phenotypic plasticity of TAMs provide opportunities to modulate TAM polarisation using mAbs to promote anti-tumoural phenotypes. In this review, we discuss the role of TAMs in ovarian cancer TME and the emerging strategies to target the contributions of these cells in tumour progression through the rationale design of mAbs.

## Teaser

The unmet clinical need for effective treatments in ovarian cancer has yet to be addressed using monoclonal antibodies (mAbs). In this review we discuss the role of tumour-associated macrophages (TAMs) in ovarian cancer and consider how TAMs can be modulated by novel mAb therapies to provide unique opportunities for clinical efficacy.

## Introduction

Ovarian cancer has the highest mortality rate among gynaecological malignancies [1]. This poor patient prognosis may be promoted by features such as rapid peritoneal metastasis of tumours, as well as tumour resistance to both current therapies and anti-tumour immunity [2]. These features are aided by the unique tumour microenvironment (TME) in the tumour mass and intraperitoneal space of patients [2]. In addition to cancer cells, a milieu of tumour-supportive cells including tumour-associated macrophages (TAMs), mesenchymal stromal cells (MSCs), fibroblasts and adipocytes are frequently sustained within the cavity by an abnormal build-up of soluble factor-rich fluid, known as peritoneal ascites [3]. Thus, an urgent need exists to better understand this unique TME, as well as develop novel therapies which specifically target its constituents. This review focuses on the therapeutic possibilities associated with targeting TAMs using monoclonal antibody (mAb) approaches.

## Monoclonal antibodies and the challenges of developing therapies for ovarian cancer

Over the past 30 years, mAb therapies have become widely used in cancer treatment, offering significant advantages relative to conventional chemotherapy and radiotherapy, including high specificity and affinity for a single epitope target, which limits off-target effects [4]. Therapeutic antibodies can be exploited to directly block tumorigenic signalling [4]. They can also engage immune effector molecules or cells via their crystallisable fragment (Fc) regions to trigger cytotoxic effector functions and amplify anti-tumour immune responses [4]. Moreover, the last decade has witnessed a rapid expansion of mAbs targeting the inhibition of immune checkpoints, known as immune checkpoint blockade (ICB) [5]. Immune checkpoints are frequently co-opted by tumours to suppress anti-tumour immunity [5]. For example, programmed death-ligand 1 (PD-L1) can engage programmed cell death protein 1 (PD-1) on T cells to induce their functional exhaustion, and therefore ICB offers a powerful tool to unleash suppressed anti-tumour immune responses in patients [6].

Despite significant successes in other tumour types, mAb therapies in ovarian cancer frequently report disappointing clinical trial results (Table 1). In recent years, experimental mAb therapies have moved away from solely targeting ovarian tumours and instead have sought to also modulate the wider TME. For example, the sole mAb licensed in the UK for the treatment of ovarian cancer is Bevacizumab (Avastin), which targets vascular endothelial growth factor A (VEGF-A), a molecule abundantly secreted by TAMs and fibroblasts to drive neo-angiogenesis and ascites formation [7]. There is now a growing interest in targeting TAMs and TAM-derived factors with mAbs.

**Table 1.** Examples of monoclonal antibodies (mAbs) in clinical trials which target tumour-associated macrophages (TAMs) and their functions for the treatment of ovarian cancer. Antibodies are categorised by their mechanism of action. Only Phase I/II and higher trials were included. If a specific agent has progressed to Phase III trials, only Phase III trials were included for this agent. Trials were only included if ovarian cancer patients were/are eligible for enrolment.

Name	Antibody specificity and isotype	Phase	Date registered on clinicaltrials.gov	Trial design	Outcome	References
**Targeting the role of TAMs in metastasis**						
Siltuximab (Sylvant)	IL-6-specific chimeric IgG1	II	February 2009	Mixed solid tumours (35 colorectal, 29 ovarian, 9 pancreatic, and 11 other). Single treatment arm.	84 patients enrolled. 0 OR, 5 SD.	NCT00841191 [8]
		II	Not registered on clinciatrials.gov	Relapsed, progressing ovarian cancer. Single treatment arm.	18 patients enrolled. 1 PR and 7 SD.	[9]
Tocilizumab (Actemra)	IL-6R-specific humanised IgG1	I/II	July 2012	Recurrent epithelial ovarian cancer. Phase I: dose escalation of Tocilizumab during the first three cycles of carboplatin plus doxorubicin. At the highest Tocilizumab dose, Peg-Intron (Pegylated interferon α) was added. Phase II: design not stated.	Phase I: 21 patients enrolled.3 CRs, 8 PRs, 6 SD. Phase II: ongoing.	NCT01637532 [10]
Targeting the role of TAMs in neo-angiogenesis						
Bevacizumab (Avastin)	VEGF-A-specific humanised IgG1	III	July 2009	Platinum-resistant ovarian cancer. Chemotherapy (pegylated liposomal doxorubicin (PLD) and paclitaxel or topotecan) alone or combination with Bevacizumab. Randomised, open-label.	Improved PFS and ORR relative to chemotherapy alone.	NCT00976911 [11]
		III	February 2009	Recurrent, platinum-sensitive ovarian cancer. Gemcitabine and carboplatin treatment plus Bevacizumab or placebo. Randomised, double-blind.	Did not improve OS relative to placebo.	NCT00434642 [12]
		III	December 2005	Incompletely resected Stage III/IV ovarian, fallopian tube, or primary peritoneal carcinoma. 3 treatment arms: -Chemotherapy (carboplatin and paclitaxel) -Chemotherapy and concurrent bevacizumab Chemotherapy and concurrent and maintenance bevacizumab. Randomised, double-blind.	No survival differences for patients who received bevacizumab relative to chemotherapy alone.	NCT00262847 [13]
		III	November 2007	Recurrent, platinum-sensitive epithelial ovarian cancer, primary peritoneal or fallopian tube cancer. Chemotherapy (carboplatin and paclitaxel or gemcitabine) alone or in combination with Bevacizumab. Randomised, open-label.	Improved OS relative to chemotherapy alone.	NCT00565851 [14]
		III	November 2017	Recurrent ovarian, fallopian tube or primary peritoneal cancer. Bevacizumab and chemotherapy (PLD or paclitaxel) plus Atezolizumab (Tecentriq) (anti-PD-L1 mAb) or placebo. Randomised, triple-blind.	Ongoing.	NCT03353831
		III	November 2010	Ovarian, fallopian tube or primary peritoneal carcinoma. Bevacizumab and chemotherapy (carboplatin and paclitaxel) as first-line treatment. Single treatment arm.	Longest reported PFS for first-line Bevacizumab-containing therapy.	NCT01239732 [15]
		III	March 2013	Recurrent, platinum-sensitive ovarian cancer. Second-line chemotherapy (PLD or gemcitabine or paclitaxel) alone or in combination with Bevacizumab after Bevacizumab/chemotherapy first-line. Randomised, open-label.	Improved PFS relative to chemotherapy alone.	NCT01802749 [16]
		III	November 2018	Newly diagnosed advanced ovarian cancer. Durvalumab (Imfinzi) (anti-PD-1 mAb) in combination with platinum-based chemotherapy and Bevacizumab followed by maintenance Durvalumab and Bevacizumab or Durvalumab, Bevacizumab and Olaparib (poly ADP ribose polymerase (PARP) inhibitor). Randomised, double-blind.	Ongoing.	NCT03737643
		III	August 2021	Newly diagnosed advanced ovarian cancer. Chemotherapy (carboplatin and paclitaxel) followed by maintenance Niraparib (PARP inhibitor) alone or in combination with concurrent and maintenance Bevacizumab. Randomised, open-label.	Ongoing.	NCT05009082
		III	September 2016	Recurrent, platinum-sensitive ovarian, primary peritoneal or fallopian tube carcinoma. Atezolizumab (anti-PD-L1 mAb) in combination with platinum-based chemotherapy plus concurrent and maintenance bevacizumab. Randomised, triple-blind.	Ongoing.	NCT02891824
		III	June 2007	Newly diagnosed ovarian, primary peritoneal or fallopian tube carcinoma. Chemotherapy (carboplatin and paclitaxel) alone or in combination with Bevacizumab. Randomised, open-label.	Improved PFS.	NCT00483782 [17]
		III	December 2015	Platinum-resistant ovarian, primary peritoneal or fallopian tube. Chemotherapy and Bevacizumab alone or in combination with combretastatin A-4 phosphate (CA4P) (Neo-vasculature-targeting agent). Randomised, quadruple-blind.	Terminated (interim analysis failed to show efficacy benefit).	NCT02641639
		III	August 2009	Stage II-III ovarian carcinoma, fallopian tube or primary peritoneal cancer. Bevacizumab in combination with intravenous or intraperitoneal chemotherapy. Randomised, open-label.	No improvement in PFS for intraperitoneal chemotherapy relative to intravenous chemotherapy.	NCT00951496 [18]
		III	August 2018	Untreated stage III/IV ovarian, fallopian tube, primary peritoneal cancer. Chemotherapy (paclitaxel and carboplatin) plus Bevacizumab or placebo. Randomised, double-blind.	Ongoing.	NCT03635489
		III	January 2018	Stage III/IV ovarian, fallopian tube, primary peritoneal cancer with post-operative macroscopic disease or who will receive neoadjuvant therapy followed by interval surgery. Chemotherapy (paclitaxel and carboplatin) and Bevacizumab with Atezolizumab (anti-PD-L1 mAb) or placebo. Randomised, double-blind.	Ongoing. Interim results: No improvement in OS relative to placebo.	NCT03038100 [19]
		II/III	July 2016	Platinum-resistant ovarian cancer. 3 treatment arms: -PLD and Atezolizumab (anti-PD-L1 mAb) -PLD and Bevacizumab -PLD and Atezolizumab and Bevacizumab. Randomised, open-label.	Ongoing.	NCT02839707
		III	July 2015	Stage IIIB/IV high-grade serous or endometroid ovarian, fallopian tube or primary peritoneal cancer which has responded to first-line chemotherapy and Bevacizumab. Maintenance Bevacizumab plus Olaparib (PARP inhibitor) or placebo. Randomised, double-blind.	Improved PFS relative to placebo.	NCT02477644 [20]
		III	July 2020	Hormone-receptor-positive high-grade serous or endometrioid ovarian carcinoma. Chemotherapy (paclitaxel and carboplatin) and Bevacizumab plus Exemestane (Aromasin) (Aromatase inhibitor) or placebo. Randomised, quadruple-blind.	Ongoing.	NCT04460807
Ramucirumab	VEGFR-2-specific human IgG1	II	July 2008	Recurrent ovarian, fallopian tube, or primary peritoneal carcinoma. Single treatment arm.	Did not meet pre-determined efficacy endpoints (PFS at 6 months and ORR).	NCT00721162 [21]
Trebananib	Ang1/2-specific peptide-Fc fusion protein	III	January 2011	Recurrent ovarian cancer. PLD plus Trebananib or placebo. Randomised, double-blind.	Improved ORR and duration of response (DOR) relative to placebo. No improvement in PFS.	NCT01281254 [22]
		III	September 2010	Recurrent ovarian carcinoma, primary peritoneal cancer or fallopian tube cancer. Paclitaxel plus Trebananib or placebo. Randomised, double-blind.	Improved PFS relative to placebo. Improved OS in patients with ascites at baseline.	NCT01204749 [23]
		III	January 2012	Recurrent ovarian carcinoma, primary peritoneal cancer or ­fallopian tube cancer. Chemotherapy (paclitaxel and carboplatin) plus Trebananib or placebo. Randomised, triple-blind.	Did not improve PFS relative to placebo.	NCT01493505 [24]
**Targeting TAM immunomodulation**						
ABBV-428	CD40/mesothelin-bispecific mAb	I/II	November 2016	Ovarian cancer and mesothelioma. Single treatment arm.	25 patients enrolled. 0 OR.	NCT02955251 [25]
**Triggering Fc-mediated functions of TAMs**						
Anti-CD47 mAb (specific mAb identity not provided)	CD47-specific mAb (specific mAb identity not provided)	I/II	October 2020	Unresectable/metastatic solid tumours (including ovarian cancer). SHR2150 (TLR7 agonist) and chemotherapy plus anti-PD1 mAb or anti-CD47 mAb (specific mAb identities not provided). Non-randomised, open-label.	Recruiting.	NCT04588324
AO-176	CD47-specific humanised IgG2	I/II	February 2019	Advanced solid tumours for which standard therapy is no longer effective or does not exist (including ovarian cancer). AO-176 alone or in combination with paclitaxel or Pembrolizumab (anti-PD1 mAb). Non-randomised, open-label.	Recruiting. Interim results: 27 enrolled. 1 PR (endometrial carcinoma), 7 SD.	NCT03834948 [26]
Catumaxomab (Removab)	EpCAM and CD3 bi-specific mAb	II/III	February 2009	EpCAM+ (Epithelial Cell Adhesion Molecule) cancer with symptomatic malignant ascites. Paracentesis alone or in combination with Catumaxomab. Randomised, open-label.	Prolonged puncture-free survival and time to next paracentesis relative to paracentesis alone	NCT00836654 [27]
Farletuzumab	FRα-specific humanised IgG1	III	February 2009	Platinum-sensitive ovarian cancer in the first relapse. Chemotherapy (carboplatin and taxanes) plus Farletuzumab or placebo. Randomised, quadruple-blind.	No improvement in PFS relative to placebo. Improved PFS in subgroup with lower CA125 levels.	NCT00849667 [28]
Trastuzumab (Herceptin)	HER2-specific humanised IgG1	II	January 2003	Early relapse (<6 months) ovarian cancer overexpressing HER2, previously treated with carboplatin and paclitaxel. Chemotherapy (carboplatin and paclitaxel) alone in ­combination with Trastuzumab. Non-randomised, open-label.	41 patients enrolled. ORR of 7.3%, 1 CR and 2 PRs.	NCT00189579 [29]
Pertuzumab (Perjeta)	HER2-specific humanised IgG1	III	September 2012	Recurrent, platinum-resistant ovarian carcinoma with low HER3 mRNA expression. Chemotherapy (topotecan, paclitaxel or gemcitabine) with Pertuzumab or placebo. Randomised, double-blind.	No improvement in OS relative to placebo.	NCT01684878
Panitumumab (Vectibix)	EGFR-specific human IgG2	II	March 2009	Platinum-resistant, KRAS(Kirsten rat sarcoma virus) wild-type ovarian cancer. Chemotherapy (PDL) with panitumumab. Single treatment arm.	Completed (no results reported).	NCT00861120
		II	July 2011	Recurrent, platinum-sensitive, KRAS wild-type recurrent ovarian cancer. Chemotherapy (carboplatin and PLD or gemcitabine) alone in combination with Panitumumab. Randomised, open-label.	No improvement in PFS relative to chemotherapy alone.	NCT01388621 [30]
Cetuximab (Erbitux)	EGFR-specific chimeric IgG1	II	May 2004	Recurrent ovarian or primary peritoneal carcinoma. Single treatment arm.	Terminated (due to lack of sufficient efficacy).	NCT00082212
		II	July 2004	Advanced stage ovarian, primary peritoneal or fallopian tube cancer. Chemotherapy (paclitaxel and carboplatin) with Cetuximab. Single treatment arm.	Completed (no results reported).	NCT00086892
		II	June 2003	Newly diagnosed advanced-stage ovarian, primary peritoneal, or fallopian tube cancer. Chemotherapy (paclitaxel and carboplatin) with Cetuximab. Single treatment arm.	Completed (no results reported).	NCT00063401

## TAMs in ovarian cancer

Macrophages are highly abundant mononuclear phagocytic cells present in almost every human tissue [31].

Macrophages are both important for inflammatory reactions and homeostatic functions. Monocyte-derived macrophages (MDMs) rapidly increase in number during inflammatory events such as infection, to aid the restoration of homeostasis through the promotion of pathogen clearance and subsequently tissue repair [32]. Moreover, tissue-resident macrophage (TRM) populations, constituted by both self-renewing pre-natal derived macrophages and short-lived adult-derived MDMs, promote the maintenance of homeostatic tissue function in the absence of inflammation [31, 33]. For example, TRMs are key in the regulation of vascular integrity, folliculogenesis, and ovulation in the ovaries [34, 35].

TAMs frequently constitute a highly abundant population within TMEs, typified in ovarian cancer, where they can account for over 50% of all cells in peritoneal tumours and ascites [36]. Initially, macrophages were thought to be strictly anti-tumoural, capable of phagocytosing malignant cells and amplifying anti-tumour immunity [37]. However, although TAM density may constitute a positive prognostic factor in colorectal cancer, in most other malignancies it is negatively associated with patient outcomes [38–43].

**Fig. 1:**
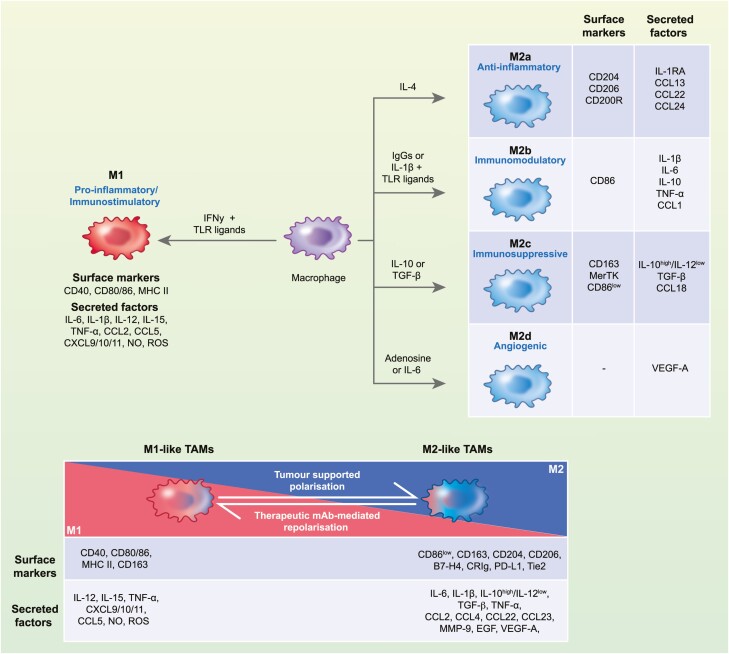
Phenotypic polarization of macrophages and TAMs in ovarian cancer. (a) *In vitro* polarization of human macrophages by different stimuli can generate phenotypically distinct subsets. These include pro-inflammatory and immunostimulatory M1, and a range of M2-associated subsets (M2a–d), each exhibiting enrichment for a specific M2-associated anti-inflammatory and pro-repair activity. However, these polarisation states are increasingly being regarded as a spectrum, with chimeric M1/M2 subsets more commonly being identified *in vivo*. This M1/M2 polarization spectrum is exhibited in TMEs, with TAMs frequently being categorised into M1-like and M2-like TAMs. The TME in ovarian cancer drives macrophage polarisation towards the M2 end of the spectrum, resulting in predominance of M2-like macrophages. These TAMs are characterised by primarily M2-associated immunosuppressive and pro-repair function, as well as by M1-associated pro-tumoural inflammatory functions. Amongst mAb therapies targeting TAMs in ovarian cancer, a prevalent aim is to shift the prevailing TAM phenotype towards M1-like TAMs, which primarily exhibit M1-associated anti-tumoural immunostimulatory function. NO, nitric oxide; ROS, reactive oxygen species; TLR, toll-like receptor; MMP, matrix metalloproteinases; MHC II, major histocompatibility complex class II; IL-1RA, interleukin-1 receptor antagonist; TGF-β, transforming growth factor-β; EGF, epidermal growth factor.

In ovarian cancer, total TAM density exhibits no prognostic significance [44, 45]. However, stratification of patients according to specific macrophage subsets based on traditional M1 and M2 polarity has revealed paradoxical associations with survival. The M1/M2 model represents a highly simplified description of macrophage phenotypes, defined as pro-inflammatory and immunostimulatory (M1) and immunosuppressive and pro-repair (M2) phenotypes [46] (Figure 1). One study found that tumour density of TAMs expressing M2 marker CD163 negatively correlated with patient overall survival (OS) [47]. Furthermore, a recent study that examined a subset of M1 (human leukocyte antigen DR (HLA-DR) and inducible nitric oxide synthase (iNOS)) and M2 (CD163 and VEGF-A) markers found that a high M1/M2 ratio was associated with improved survival, when present intra-tumourally, but not in the tumour stroma [48].

**Fig. 2:**
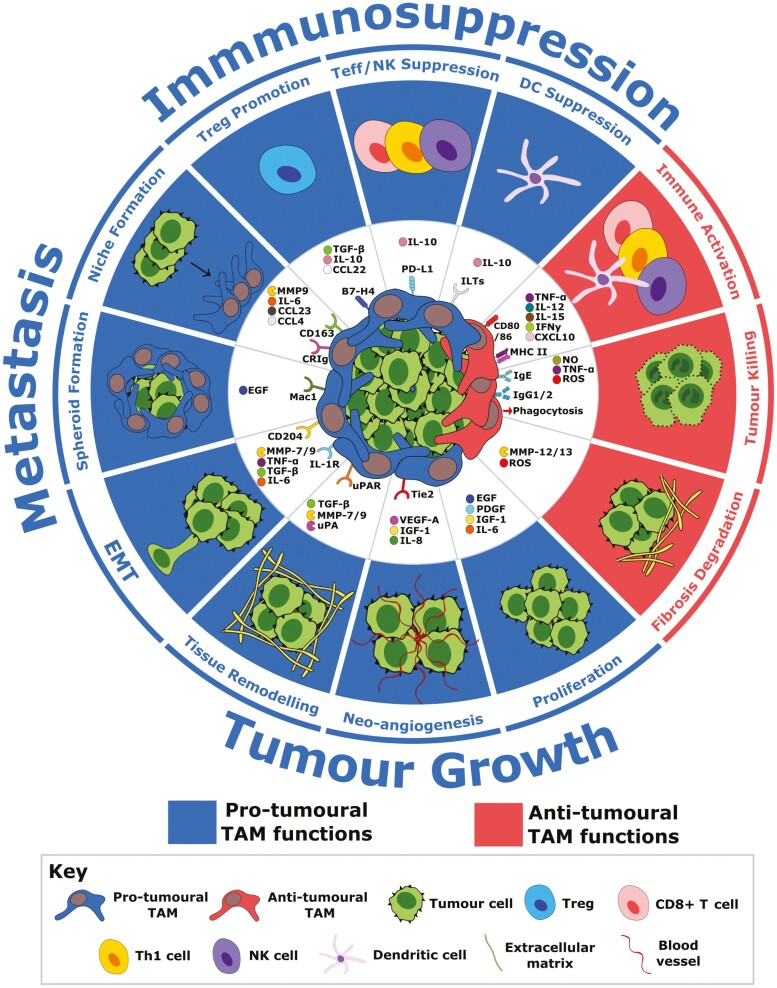
Pro-and anti-tumoural functions of tumour-associated macrophages (TAMs) in ovarian cancer. The pro- and anti-tumoural processes promoted by TAMs in ovarian cancer and the cell surface receptors and ligands and secreted factors that mediate these activities. Treg, regulatory T cell; Teff, effector T cell; NK, natural killer cell; DC, dendritic cell; NO, nitric oxide; ROS, reactive oxygen species; uPAR, urokinase plasminogen activator surface receptor; uPA, urokinase plasminogen activator; MMP, matrix metalloproteinases; PDGF, platelet-derived growth factor; IGF-1, insulin-like growth factor 1; Mac1, macrophage-1 antigen; ILT, Ig-like transcript; TGF-β, transforming growth factor-β; MHC II, major histocompatibility complex class II.

*In vivo*, especially in TMEs, the binary M1/M2 model has proven oversimplified, with a spectrum model possibly offering a more accurate representation of macrophage polarisation [46]. By this model, polarisation can produce a broader range of distinct M1 and M2 macrophage subsets, such as M2a-d, as well as a range of subsets with chimeric M1/M2 features (Figure 1). TAMs can exhibit this M1/M2 chimerism and consequently are frequently referred to as M1- and M2-like [49]. In most tumour types, including ovarian cancer, M2-like TAMs predominate [50–52]. M2-like denotes a population-level phenotype that is skewed towards immunosuppressive and pro-repair functions, but which also exhibits some M1 properties that confer inflammatory pro-tumoural effects [51, 53]. Consequently, in the ovarian TME, both pro- and anti-inflammatory TAM activity has been demonstrated to support pro-tumoural processes, including tumour growth, metastasis, neo-angiogenesis, therapy resistance, and immunosuppression (Figure 2) [51, 54–57].

A substantial investigation has been undertaken into the complex interactions of TAMs with ovarian tumours, to identify specific subsets and TAM-derived molecules which promote tumour progression and therefore may represent novel targets for experimental mAb therapies. Specifically, a prevalent aim amongst TAM-targeting mAbs is to shift the prevailing TAM phenotype away from M2-like immunosuppressive towards immunostimulatory M1-like properties (Figure 1).

## Depletion of TAMs via mAbs

In view of the extensive pro-tumoural activity displayed by TAMs in ovarian cancer, several mAb therapies have sought to deplete their presence in the TME.

Firstly, C–C motif chemokine ligand 2 (CCL2) drives recruitment of monocytes expressing C–C motif chemokine receptor 2 (CCR2) from the blood into the ovarian TME, with densities of intra-tumoural TAMs and cell expressing CCL2 positively correlating [58, 59]. CCL2 is frequently highly abundant in the tumour parenchyma, stroma, and ascites and is secreted by both tumour cells and supporting cells such as TAMs themselves, to drive a positive recruitment feedback loop [58, 59]. Secondly, colony-stimulating factor 1 (CSF-1) is a haematopoietic growth factor that binds to colony-stimulating factor 1 receptor (CSF-1R) expressed on monocytes and macrophages [60]. In addition to acting as a chemokine for monocytes, CSF-1 also promotes their survival and differentiation into TAMs, generating a phenotype that is skewed towards M2-associated immunosuppressive activity, in the absence of additional signals [50]. In ovarian cancer, high levels of CSF-1 in the serum and ascites are associated with poorer patient outcomes [61, 62]. In one cohort of patients, co-expression of CSF-1 and CSF-1R in ovarian metastatic lesions was associated with decreased progression-free survival (PFS) [63].

**Fig. 3:**
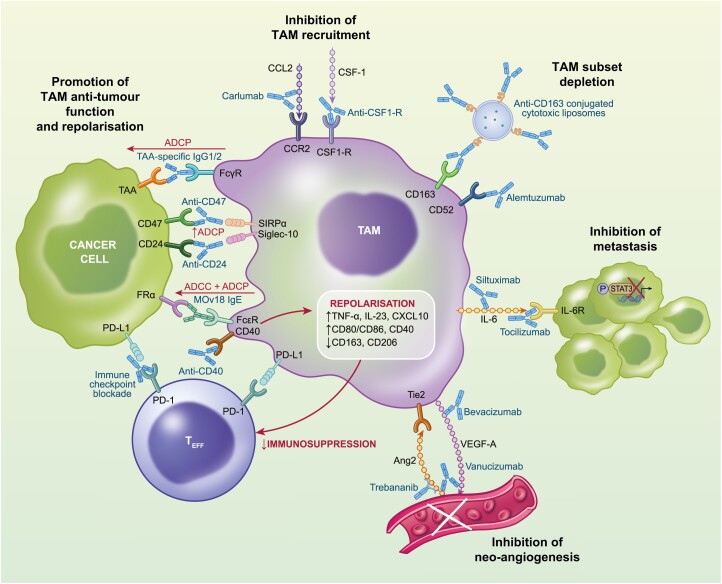
Therapeutic targeting of tumour-associated macrophages (TAMs) in ovarian cancer by monoclonal antibodies (mAbs). Summary of the mAb strategies which target ovarian cancer TAMs and their functions, and their mechanism of action. Teff, effector T cell; ADCC, antibody-dependent cellular cytotoxicity; ADCP, antibody-dependent cellular phagocytosis; TAA, tumour-associated antigen; FRα, folate receptor alpha.

mAbs targeted against CCL2 and CSF-1R to reduce TAM recruitment and/or survival have been investigated in phase I clinical trials involving ovarian cancer patients (Figure 3) (NCT02526017) [64]. With respect to anti-CCL2 mAbs, in an ovarian cancer xenograft mouse model, the anti-human CCL2 IgG1 human mAb Carlumab was found to enhance the efficacy of chemotherapies paclitaxel and carboplatin [65]. In a phase I clinical trial involving eight patients with ovarian cancer of the 44 enrolled, Carlumab was well tolerated; however, it did not induce any patient objective responses (ORs) [64]. Carlumab proceeded to a Phase II study in castration-resistant prostate cancer, where again no ORs were observed, leading to its discontinuation [66].

Several reasons have been cited for the lack of efficacy of anti-CCL2 mAbs. Firstly, clinical trials suggest a failure to durably neutralise CCL2 levels in patient sera [66]. Secondly, in the event of a successful CCR2/CCL2 blockade, this would indiscriminately inhibit the contribution of MDMs to the TAM pool only. Consequently, it would firstly impede the development of potential anti-tumoural MDM TAM subsets and secondly, would have a minimal effect on pro-tumoural TRM subsets. For example, in a syngeneic ovarian cancer mouse model, whilst CD163+ Tim4+ TRMs were found to be indispensable for tumour progression, *Ccr2*^*−/−*^ mice exhibited unperturbed disease [55].

In ovarian cancer preclinical models, CSF-1/CSF-1-R blockade has only been investigated via small-molecule inhibitors as opposed to mAbs. However, in a syngeneic ovarian cancer mouse model, a combination of CSF-1 inhibition with chemotherapy docetaxel reduced tumour lung metastasis [67]. Crucially both TAM abundance and TME expression of M2-associated TAM genes *ARG1*, *MRC1*, and *IL10* were reduced, concurrent with an increase in CD8+ T cell tumour infiltration, suggesting a preferential targeting of M2-associated immunosuppressive TAM subsets.

However, across cancer types, clinical trials investigating anti-CSF-1R mAbs have reported disappointing results. Although multiple experimental mAbs have shown evidence of target specificity, characterised by increased serum CSF-1 and reductions in M2-associated CD163+, CD206+, and CSF-1R+ TAMs, insufficient anti-tumour activity has been displayed [68, 69]. Despite possible preferential M2-like macrophage depletion, CSF1/CSF1-R blockade still appears too indiscriminate in terms of TAM inhibition. Due to the spectral nature of TAM polarisation, M1-associated functions which can support anti-tumour immunity may also be collaterally lost (Figure 2). In a Lewis lung carcinoma (LLC) mouse model, CSF-1/CSF-1R blockade was found to deplete intra-tumoural NK cells and increase tumour metastasis, due to a loss of the TAM-derived NK survival factor IL-15 [70].

Consequently, emerging strategies have targeted subset-level TAM depletion. This approach has proven efficacious in preclinical models, with depletion of the murine CD163+ Tim4+ TRM subset via anti-CD163 mAb-coated cytotoxic liposomes found to reduce ovarian tumour burden in mice [55]. Although TAM markers can vary between mouse and human, a recent study identified a human TAM population homologous to murine Tim4+ TAMs, characterised by complement receptor of the immunoglobulin superfamily (CRIg) expression [71]. This provides hope that clinical investigation of such a targeted approach may soon be possible (Figure 3).

## mAbs targeting the role of TAMs in tumour metastasis

One of the key factors in the poor prognoses of ovarian cancer patients is the propensity of tumours to undergo peritoneal metastasis early in tumourigenesis. Ovarian peritoneal metastasis is a complex, multi-step process and mAb targets for this process are currently limited. However, one emerging target is IL-6, which activates signal transducer and activator of transcription 3 (STAT3) signalling within tumour cells [9]. STAT3 activation has been demonstrated to contribute to each step in the metastatic cascade and be promoted by TAMs (Figure 2). Firstly, TAM-secreted IL-6 promotes epithelial–mesenchymal transition (EMT) in cancer cells to induce their shedding from the primary tumour [51, 72, 73] (Figure 2). Subsequently, cancer cells migrate across the intraperitoneal space as multi-cellular spheroids containing supporting cells such as TAMs and fibroblasts [54, 74]. Following spheroid implantation into the cavity wall, TAM-induction of STAT3 activation promotes spheroid disaggregation and spreading to further sites [72]. IL-6/STAT3 activation also induces cancer stem cell (CSC) formation in metastatic lesions, which promotes therapeutic resistance [55, 75].

IL-6 expression in ovarian cancer patient tumours and serum both increase with disease stage, whilst high tumour IL-6 levels inversely correlate with patient survival [9, 75]. Moreover, expression of M2-associated TAM marker CD163 is associated with both ascites levels of IL-6 and reduced patient relapse-free survival (RFS), whilst TAMs have been identified as the highest secretors of IL-6 in the TME [51, 76].

Siltuximab (Sylvant) and Tocilizumab (Actemra) are IgG1 mAbs targeted against IL-6 and IL-6 receptor (IL-6R), respectively, to induce a blockade of IL-6-mediated STAT3 activation (Figure 3) [77]. In ovarian cancer patients, both mAbs have shown good tolerability profiles and effective IL-6/IL-6R blockade, characterised by decreased serum C-reactive protein (CRP) and STAT3 activation and increased serum IL-6 and soluble IL-6R [8–10]. However, despite reducing serum levels of IL-6 regulated cytokines involved in the metastatic cascade, such as C–X–C motif chemokine ligand 12 (CXCL12), VEGF-A and CCL2, Siltuximab has hitherto not shown clear efficacy in its two-phase II trials [8, 9] (Table 1). However, in both trials, ovarian cancer patient numbers were low, and some level of activity was still shown; with one partial response (PR) and seven patients displaying stable disease (SD), from 18 ovarian cancer patients in one trial [9]. Moreover, all recruited patients had a late-stage disease and therefore it is possible that the anti-metastatic effects of IL-6/IL-6R blockade would be stronger in combination with a cytotoxic therapy such as chemotherapy, to prevent the outgrowth of new metastatic lesions following chemotherapy-induced tumour regression. Accordingly, in the sole phase I trial of Tocilizumab in ovarian cancer patients, the mAb was investigated in combination with chemotherapies carboplatin and doxorubicin and displayed evidence of a survival benefit, with three complete responses (CRs), eight PRs, and six SD out of 21 patients on the trial (Table 1) [10]. These findings underline the merit of assessing IL-6/IL-6R blockade in larger, randomised studies, especially in combination with cytotoxic therapies.

## mAbs targeting the role of TAMs in tumour neo-angiogenesis

A key requirement for the development of both the primary and metastatic tumours is the establishment of access to the circulatory system via neo-angiogenesis. TAMs potently promote neo-angiogenesis, displaying enrichment at sites with high angiogenic requirements, including hypoxic tumour nests, nascent peritoneal tumours, and perivascular regions [54, 56, 58, 78, 79] (Figure 2). A key neo-angiogenic factor is VEGF-A which binds to vascular endothelial growth factor receptors 1, 2 (VEGFR 1,2) on endothelial cells, triggering vessel development [80]. VEGF-A is highly upregulated in ovarian cancer on tumour cells, MSCs and TAMs, with high patient VEGF-A serum levels associated with increased micro-vessel density and ascites levels and decreased survival [54, 81–85]. Moreover, depletion of peritoneal TAMs in a syngeneic ovarian cancer mouse model was found to reduce ascites formation and peritoneal metastasis, concurrent with a reduction of ascitic VEGF-A [86].

The IgG1 mAb Bevacizumab blockades VEGF-A/VEGFR-mediated neo-angiogenesis through binding to VEGF-A (Figure 3) [87]. Bevacizumab is the only mAb licensed in the UK for the treatment of ovarian cancer [7]. It is currently recommended as maintenance therapy following first-line chemotherapy, to inhibit tumour recurrence [88]. However, eventually most patients develop resistance to Bevacizumab treatment [89, 90].

TAMs may play a significant role in this resistance. In a murine model of Bevacizumab-resistant ovarian cancer, tumours exhibited restored response when treated with a TAM-depleting anti-CSF-1 mAb [91]. Specifically, VEGF-A/VEGFR blockade is considered to enhance tumour hypoxia, which induces chemoattraction of pro-angiogenic TAM subsets to restore neo-angiogenesis [92]. A key chemoattraction pathway may be via angiopoietin-2 (Ang2) engagement of its receptor Tie2 on TAMs. In ovarian cancer patients, Tie2+ TAM density positively correlates with micro-vessel density [56]. This underlines the limitations of therapeutically targeting a single pro-tumoural mediator, such as VEGF-A, due to redundancy within the TME. Consequently, experimental strategies have sought to concurrently neutralise both the VEGF-A/VEGFR and Ang2/Tie2 pathways (Figure 3). For example, in a VEGF-A/VEGFR blockade-resistant syngeneic pancreatic cancer mouse model, concurrent mAb targeting of Ang2 suppressed re-vascularisation and tumour progression [93].

The potential utility of combination therapy for targeting neo-angiogenesis has been indicated clinically. Firstly, in a Phase III trial, peptide-Fc fusion protein Trebananib, which targets Ang2, as well as additional Tie2 ligand, Ang1, was found to enhance PFS in ovarian cancer patients (Table 1) [23]. Secondly, a bispecific Ang2/VEGF-A-targeting mAb, Vanucizumab, underwent a Phase I trial in ovarian cancer patients and demonstrated potential clinical activity, with 20% of patients displaying an OR (Figure 3) [94].

Due to the redundancy of neo-angiogenesis pathways and the centrality of TAMs in driving them, a more durable therapeutic strategy may be to specifically target pro-angiogenic TAM subsets via mAbs (Figures 2 and 3). This could be achieved through identification of a surface marker selectively expressed on a TAM subset co-expressing VEGF-A and Tie2, to achieve selective depletion, without inhibiting potential anti-tumoural TAM subsets. For example, Tie2+ TAMs isolated from ovarian cancer patient ascites express high levels of CD52, the target of licensed IgG1 mAb Alemtuzumab (Lemtrada) [79]. In a syngeneic ovarian cancer mouse model, anti-CD52 treatment was demonstrated to restrict tumour neo-angiogenesis and growth, substantiating the potential clinical utility of subset-level TAM-targeting. Consequently, Alemtuzumab entered Phase I investigation in ovarian cancer patients, but disappointingly the study was terminated due to poor patient enrolment (NCT00637390).

## mAbs to trigger Fc-mediated functions of TAMs

Instead of inhibiting the pro-tumoural activity of TAMs, specific mAbs have sought to promote the anti-tumour function of TAMs.

One such approach is through an antibody’s simultaneous engagement of tumour-associated antigens (TAAs) on tumour cells and antibody Fc receptors on TAMs, to trigger tumouricidal TAM effector function [95]. This can result in the engulfment of tumour cells via antibody-dependent cellular phagocytosis (ADCP) and paracrine tumour cell lysis via antibody-dependent cellular cytotoxicity (ADCC) (Figure 3) [95]. All licensed full-length TAA-targeting mAbs are of the IgG isotype [96]. Among IgGs, IgG1 isotype features the highest affinity for all Fc-γ receptors (FcγRs), whilst IgG2 has a high affinity for FcγRIIa-H131, which is mainly expressed on macrophages [97]. Consequently, they have increasingly been recognised to involve Fc-mediated TAM effector functions as part of their mechanism of action [97].

Although TAA-targeting IgG mAbs have displayed efficacy in solid tumours, clinical results in ovarian cancer have been disappointing (Table 1). This disparity is illustrated by the anti-human epidermal growth factor receptor 2 (HER2) IgG1 mAb Trastuzumab (Herceptin). HER2 is expressed in up to 66% of ovarian tumours and negatively correlates with OS [98]. However, despite demonstrating anti-tumour efficacy in a variety of preclinical ovarian cancer models, in a Phase II trial in ovarian cancer patients with high tumour HER2 expression, only 7.3% responded to the treatment (Table 1) [29,99–101].

One emerging strategy to enhance the efficacy of TAA-targeting IgG mAbs is through mAb-mediated blockade of ‘don’t eat me’ signals expressed on tumour cells, to enhance the contribution of TAM effector functions to their mechanism of action. For example, tumour cell CD47 and CD24 engage cognate receptors on TAMs, signal regulatory protein-α (SIRPα) and sialic acid-binding Ig-like lectin 10 (Siglec-10), respectively, to trigger signalling cascades which inhibit macrophage phagocytosis [102, 103] (Figure 3). CD47 expression is predictive of disease stage and prognosis in patients with ovarian cancer, whilst one study identified ovarian tumours to express the highest level of CD24 of all cancer types analysed, with its expression inversely correlating with patient RFS [102, 104].

Pre-clinically, neutralising anti-CD47 and anti-CD24 mAbs have demonstrated enhancement of non-specific macrophage phagocytosis of ovarian cancer cells *in vitro*, as well as inhibition of tumour growth in murine ovarian cancer models [102, 105]. *In vitro*, dual blockade of CD47 and CD24 exhibited an additive effect on ovarian cancer cell phagocytosis [102]. Moreover, in other cancer types, anti-CD47 blockade has been demonstrated to enhance macrophage ADCP of tumour cells in response to TAA-targeting IgG mAbs, including Trastuzumab, anti-CD20 IgG1 Rituximab (Rituxan), and anti-epidermal growth factor receptor (EGFR) IgG1 Cetuximab (Erbitux) [106]. Furthermore, in a syngeneic breast cancer mouse model, co-treatment with Trastuzumab and anti-CD47 mAbs improved tumour control and was associated with macrophage pro-inflammatory polarisation and cytotoxic gene expression in CD8+ T cells [107].

Neutralising anti-CD47 mAbs have recently begun early phase clinical investigation across different cancer types. Interim results from a Phase I/II trial of anti-CD47 IgG2 mAb AO-176 demonstrated a good tolerability profile and possible anti-tumour activity, with 1 PR and 7 SD from 27 patients with advanced solid tumours (Table 1) [26].

Moreover, in view of the enhanced pre-clinical efficacy of TAA-targeting mAbs when in combination with anti-CD47 mAbs, several trials are currently underway using this regimen in non-ovarian solid tumours. For example, a phase II study in colorectal cancer patients of anti-CD47 IgG4 mAb Hu5F9-G4 in combination with Cetuximab, reported stable disease in 45% of patients, as well as increased macrophage tumour infiltration [108]. It remains to be seen whether the possible enhancement of TAM effector function offered by such a combination therapy could overcome the efficacy threshold that has hitherto eluded TAA-targeting IgGs in ovarian cancer.

## mAbs targeting the role of TAMs in tumour immunomodulation

Ovarian cancer patients frequently exhibit evidence of immunogenic tumour recognition, such as CD8+ T cell tumour infiltration; however immunosuppression within the TME frequently prevents their effector function [57, 109]. TAMs play a major role in this immunosuppression (Figures 2 and 3). For example, TAMs display poor upregulation of T cell co-stimulatory molecules CD80 and CD86 and an IL-10^high^IL-12^low^ secretome (Figure 2) [50]. Furthermore, they exhibit high expression of immune checkpoint ligand B7-H4, which engages T cells to suppress their proliferation [110]. High B7-H4 expression by TAMs is associated with poorer survival in patients, as well as a high density of regulatory T cells (Tregs) [110]. TAMs have been demonstrated to drive such high Treg densities through secretion of M2-associated chemokine CCL22, and once recruited, Tregs amplify this immunosuppressive axis by triggering further TAM B7-H4 enrichment and CD86 and IL-12 suppression [110, 111].

Following the disappointing clinical results of TAM depletion strategies and the difficulty of selective TAM subset depletion, recent strategies have instead sought to exploit TAM plasticity through targeting these immunosuppressive subsets by repolarisation towards an immunostimulatory, anti-tumoural phenotype (Figure 1 and 2). Among TAM repolarisation strategies, mAbs targeting the TAM co-stimulatory molecule CD40 has been the focus of clinical investigation in ovarian cancer. During type I immune responses to pathogens, CD40 agonism by Th1 cell CD40 ligand (CD40L) drives macrophages to a hyper-inflammatory phenotype [112]. Agonistic anti-CD40 mAbs aim to recapitulate this hyper-inflammatory state in TAMs (Figure 3).

Pre-clinically, anti-CD40 mAbs have predominantly been investigated in other malignant diseases; however, in an ovarian cancer xenograft mouse model, a recombinant CD40L inhibited tumour growth [113]. As monotherapies, anti-CD40 mAbs have demonstrated evidence of promoting a macrophage-driven shift in the immune landscape of tumours. In a syngeneic pancreatic cancer mouse model, anti-CD40 mAb treatment resulted in the intra-tumoural migration of hyperinflammatory monocytes and macrophages, which mediated tumour regression [114]. Moreover, in a Phase I trial, anti-CD40 IgG2 mAb Selicrelumab induced a CR in a patient with metastatic melanoma, which was sustained 9 years after therapy initiation [115]. The patient exhibited a pro-inflammatory shift in the TME post-treatment, including tumour necrosis factor (*TNF*) upregulation and downregulation of M2-associated *CSF1R*. However, a Phase I/II trial of ABBV-428, a bispecific mAb targeting CD40 (as an agonist) and tumour cell marker mesothelin, was not able to replicate these promising anti-tumoural effects in ovarian cancer patients [25].

Such clinical results have generally shifted the outlook on anti-CD40 mAbs towards combined treatment regimens with cytotoxic therapies. Firstly, chemotherapy promotes the release of TAAs during a process known as ‘immunogenic cell death’, which facilitates the generation of *de novo* T cell immunity [116]. This can be sustained as a memory T cell response in combination with anti-CD40 repolarisation in a syngeneic pancreatic cancer mouse model, for which anti-CD40 monotherapy is insufficient [117]. In a Phase I trial of Selicrelumab in combination with paclitaxel and carboplatin in patients with advanced solid tumours, including ovarian cancer, preliminary evidence of anti-tumour activity was observed, with 20% of patients displaying PRs [118].

Secondly, evidence has emerged that TAM-mediated immunosuppression underpins the lack of efficacy displayed by ovarian cancer patients in response to ICB, and therefore ICB in combination with anti-CD40 treatment has been advanced as a potentially efficacious strategy (Figure 3) [57]. Despite revolutionising therapeutic approaches in cancer types such as melanoma, ovarian cancer is one of the few malignancies in which ICB displays minimal activity [119]. One study found that ovarian cancer patient CD8+ T cells were responsive to anti-PD-1 activation at primary tumours only [57]. At metastatic sites, CD163+ TAMs were inversely associated with CD8+ T cell effector function and patient OS.

Clinically, a Phase I trial using anti-CD40 IgG2 mAb CDX-1140 with anti-PD-1 IgG4 mAb Pembrolizumab (Keytruda) is currently recruiting ovarian cancer patients (NCT03329950). This will provide the first insight into whether anti-CD40 mediated TAM repolarisation can skew the ovarian TME immune landscape towards ICB efficacy (Figure 3).

## IgE antibodies can exert macrophage-mediated anti-tumour activity by both tumour killing and pro-inflammatory activation

Currently, all licensed full-length mAbs are of the IgG isotype [96]. IgE antibodies can induce a rapid and potent skewing of the immune landscape in both allergy and parasite clearance, by distinct mechanisms to those of IgG isotypes [120, 121]. IgE antibodies exhibit unique features, relative to IgG, which may be of utility in cancer treatment via mAbs. Whilst the high-affinity IgG receptor, FcγRI, exhibits a *K*_a_ for IgG of 10^8^–10^9^ M^−1^, the high-affinity IgE receptor, Fc ε receptor I (FcεRI), exhibits a substantially higher affinity for IgE (*K*_a_ = 10^10^–10^11^ M^−1^) [122, 123]. This strong interaction may trigger rapid IgE engagement by circulating FcεRI-expressing immune cells, such as monocytes, to traffic IgE efficiently to the TME [121, 124]. Moreover, comparatively fewer Fc receptor:antibody molecules are required to be cross-linked by antigen for effector cell activation [125]. Furthermore, IgE is not subject to inhibition via an inhibitory receptor or another isotype subclass as IgG is via FcγRIIb and IgG4, respectively [126, 127]. Accordingly, there is growing evidence for the role of IgE antibodies in anti-tumour immunosurveillance, including an inverse association between serum IgE levels and the risk of gynaecological cancers including ovarian cancer [128]. Consequently, it is hypothesised that these features can be harnessed by IgE isotype mAbs to engage a unique anti-tumour immune axis in cancer patients.

Among several developed IgE mAbs targeting TAAs, MOv18 IgE, represents the first-in-class to enter clinical testing, in an ongoing phase I trial involving predominantly ovarian cancer patients (NCT02546921). MOv18 IgE is targeted against TAA, folate receptor α (FRα), which is overexpressed in 82% of serous epithelial ovarian cancers and inversely correlated with patient OS and disease-free interval (DFI) [129, 130].

In pre-clinical models, MOv18 IgE exerted anti-tumoural effects via Fc engagement of monocytes and macrophages in a two-armed mechanism, comprising tumour killing and crucially, pro-inflammatory polarisation [131]. In an ovarian cancer patient-derived xenograft (PDX) mouse model, MOv18 IgE produced superior mouse survival compared with the equivalent IgG1 [132]. This survival benefit was abrogated following depletion of monocytes from the human PBMC infusion. Furthermore, in a syngeneic rat lung metastases model, rat MOv18 IgE restricted lung metastases growth compared with its IgG2b equivalent [133]. This effect was associated with enhanced intra-tumoural macrophage migration.

*In vitro* interrogation of tumour cell killing determined that MOv18 IgE predominantly induces cancer cell ADCC by monocytes, as well as ADCP, when monocytes are IL-4-stimulated to induce expression of low-affinity IgE receptor, CD23 [132]. In comparison, the equivalent IgG1 mediated ADCP only, suggesting alternative and potentially synergistic killing mechanisms between IgE and IgG1 (Figure 3).

Consistently, *in vitro* and *in vivo* studies have found that cross-linking of TAA-bound IgE initiates a TNF-α/CCL2-mediated monocyte and macrophage pro-inflammatory recruitment feedback loop, to potentiate tumour killing and drive pro-inflammatory polarisation [133–135]. Exploration of this phenotypic shift found that cross-linking of MOv18 IgE on human monocytes by ovarian cancer cells, induced a pro-inflammatory secretome (TNF-α, CCL2, IL-10, CXCL-10, IL-1β, IL-6, and IL-23) [134]. Consistently, monocytes also upregulated co-stimulatory molecules CD80, CD86, and CD40, whereas M2-associated scavenger receptors, CD163, CD206, and MerTK, were downregulated. High tumour expression of this secreted signature was associated with improved ovarian cancer patient survival. Accordingly, this pro-inflammatory activation was found to enable repolarisation of anti-inflammatory human MDM subsets, M0 (unpolarised) and M2 (IL-4 polarised), following *in vitro* cross-linking of the TAA-specific IgE mAb SF-25 [135].

Collectively, TAA-targeting IgE mAbs may drive a unique monocyte and macrophage polarisation signature, which combined with an alternative tumour killing mechanism and superior *in vivo* anti-tumour activity, may permit sustained efficacy, which has hitherto eluded TAA-targeting IgG mAbs in ovarian cancer. Recent interim results from the Phase I clinical trial of MOv18 IgE have demonstrated the therapy to be well tolerated and, although preliminary, anti-tumour activity was displayed by one patient [136].

## Conclusion

High frequency, broad Fc receptor expression, extensive potential pro-tumoural activities and strong phenotypic plasticity, render TAMs and their associated functions as attractive mAb targets to meet the unmet clinical need in ovarian cancer (Figure 3). Antibodies designed to broadly deplete TAMs, such as CCL2/CCR2 and CSF-1/CSF1-R neutralising mAbs have performed poorly in the clinic as single agents, characterised by both a lack of effect on specific pro-tumoural subsets and collateral inhibition of anti-tumour TAM activity. Moreover, depletion of TAMs has been observed to trigger compensatory recruitment of other innate immune cells, such as myeloid-derived suppressor cells (MDSCs), to maintain an immunosuppressive TME in the absence of TAMs [137].

Consequently, instead of viewing TAMs as a deleterious population for removal, recent experimental mAb strategies have frequently sought to exploit their extensive plasticity and immunoregulatory function via phenotypic repolarisation, to mediate a TAM-driven shift in the TME towards anti-tumour immunostimulation (Figures 1 and 3). These include agonistic anti-CD40 and IgE isotype mAbs. TAM repolarisation could be of significant interest in ovarian cancer, where immunosuppressive TAM subsets which restrict anti-tumour immunity are frequently present. Both therapies have demonstrated promising preclinical activity, as well as early signs of clinical responses. In addition to potentially alleviating immunosuppression, a population-level TAM repolarisation could collaterally inhibit wider pro-tumoural TAM activity and therefore may synergise with other promising mAb candidates, such as neutralising anti-IL6 and anti-VEGF-A, which inhibit TAM’s role in metastasis and neo-angiogenesis, respectively (Figures 2 and 3).

Overall, mAb-mediated TAM repolarisation may facilitate a therapeutic window in which the ovarian TME is biased towards anti-tumour activity. Clinical exploitation of this opportunity therefore may best be achieved through combination with established cytotoxic therapies. For example, potential synergistic anti-tumour activity between TAM repolarisation and both tumour-targeting chemotherapy and adaptive immunity-targeting ICB have begun to emerge.

Further investigation of these mAb candidates and possible combination regimens is warranted in larger clinical studies, to determine whether therapeutic targeting of TAMs may represent a viable pathway for efficacy, which has generally eluded mAbs in ovarian cancer.

## Data Availability

Not applicable as the manuscript is a review article.

## Abbreviations

ADCC: antibody-dependent cellular cytotoxicity
ADCP: antibody-dependent cellular phagocytosis
Ang2: angiopoietin-2
CCL2: C–C motif chemokine ligand 2
CCR2: C–C motif chemokine receptor 2
CD40L: CD40 ligand
CR: complete responses
CRIg: complement receptor of the immunoglobulin superfamily
CSF-1: colony stimulating factor 1
CSF-1R: colony stimulating factor 1 receptor
CXCL12: C–X–C motif chemokine ligand 12
DFI: disease-free interval
EGFR: epidermal growth factor receptor
EMT: epithelial–mesenchymal transition
Fc: crystallisable fragment
FcγR: Fc-γ receptor
FcεR: Fc-ε receptor
HLA-DR: human leukocyte antigen DR
ICB: immune checkpoint blockade
iNOS: inducible nitric oxide synthase
LLC: Lewis lung carcinoma
mAb: monoclonal antibody
MDM: monocyte-derived macrophage
MSC: mesenchymal stromal cell
PD-1: programmed cell death protein 1
PD-L1: programmed death-ligand 1
PFS: progression-free survival
PR: partial response
SD: stable disease
Siglec-10: sialic acid-binding Ig-like lectin 10
SIRPα: signal regulatory protein α
STAT3: signal transducer and activator of transcription 3
TAA: tumour-associated antigen
TAM: tumour-associated macrophage
TME: tumour microenvironment
TNFα: tumour necrosis factor α
Treg: regulatory T cell
TRM: tissue-resident macrophage
VEGF-A: vascular endothelial growth factor A
VEGFR: vascular endothelial growth factor receptor
